# Behaviour and distribution of *Aedes aegypti* mosquitoes and their relation to dengue incidence in two transmission hotspots in coastal Ecuador

**DOI:** 10.1371/journal.pntd.0010932

**Published:** 2024-04-29

**Authors:** Leonardo D. Ortega-López, Mauro Pazmiño Betancourth, Renato León, Alain Kohl, Heather M. Ferguson

**Affiliations:** 1 School of Biodiversity, One Health and Veterinary Medicine, University of Glasgow, Glasgow, United Kingdom; 2 Laboratorio de Entomología Médica & Medicina Tropical LEMMT, Colegio de Ciencias Biológicas y Ambientales COCIBA, Universidad San Francisco de Quito USFQ, Quito, Ecuador; 3 MRC-University of Glasgow Centre for Virus Research, Glasgow, United Kingdom; University of Notre Dame, UNITED STATES

## Abstract

**Background:**

Dengue (DENV) transmission is endemic throughout coastal Ecuador, showing heterogeneous incidence patterns in association with fine-scale variation in *Aedes aegypti* vector populations and other factors. Here, we investigated the impact of micro-climate and neighbourhood-level variation in urbanization on *Aedes* abundance, resting behaviour and associations with dengue incidence in two endemic areas.

**Methodology/Principal findings:**

*Aedes aegypti* were collected in Quinindé and Portoviejo, two urban cantons with hyperendemic dengue transmission in coastal Ecuador. *Aedes* vectors were sampled in and around houses within urban and peri-urban neighbourhoods at four time periods. We tested for variation in vector abundance and resting behaviour in relation to neighbourhood urbanization level and microclimatic factors. *Aedes* abundance increased towards the end of the rainy season, was significantly higher in Portoviejo than in Quinindé, and in urban than in peri-urban neighbourhoods. *Aedes* vectors were more likely to rest inside houses in Portoviejo but had similar abundance in indoor and outdoor resting collections in Quinindé. Over the study period, DENV incidence was lower in Quinindé than in Portoviejo. Relationships between weekly *Ae*. *aegypti* abundance and DENV incidence were highly variable between trapping methods; with positive associations being detected only between BG-sentinel and outdoor Prokopack collections.

**Conclusions/Significance:**

*Aedes aegypti* abundance was significantly higher in urban than peri-urban neighbourhoods, and their resting behaviour varied between study sites. This fine-scale spatial heterogeneity in *Ae*. *aegypti* abundance and behaviour could generate site-specific variation in human exposure and the effectiveness of indoor-based interventions. The trap-dependent nature of associations between *Aedes* abundance and local DENV incidence indicates further work is needed to identify robust entomological indicators of infection risk.

## Author summary

Dengue transmission is hyperendemic in coastal Ecuador. However, there is limited understanding of how exposure to *Aedes aegypti* vectors varies at the neighbourhood-level and in response to local micro-climatic conditions. Here, we investigated how the abundance and resting behaviour (use of indoor versus peri-domestic sites) of *Ae*. *aegypti* varied within and between 2 coastal urban settings, Portoviejo and Quinindé cantons. Additionally we tested for associations between *Aedes* abundance and dengue case incidence. We found that the abundance of *Ae*. *aegypti* and their preference for indoor resting sites varied between the two study sites. Vectors were more abundant in urban than in peri-urban neighbourhoods at both sites. Associations between *Ae*. *aegypti* abundance and “city-wide” dengue incidence were highly variable depending on the trapping method used; with collections made in peri-domestic settings (BG sentinel traps and Prokopack aspiration) showing a positive association with incidence in contrast to indoor Prokopack aspirations. These results emphasize the need for fine-scale vector surveillance at the neighbourhood-level to identify hotspots of *Aedes* abundance and optimize vector control strategies.

## Background

Transmission of dengue (DENV), chikungunya (CHIKV) and Zika (ZIKV) viruses places a huge public health burden on countries in the Americas [1–3]. Surveillance and control of the *Aedes aegypti* mosquito vectors that transmit these pathogens and raising public awareness have been the main control strategies [4–7]. Sustained vector surveillance is recommended to detect and respond to potential arbovirus outbreaks [7]. Surveillance is complex because *Ae*. *aegypti* populations are highly heterogeneous in time and space; with distribution and abundance associated with environmental and climatic factors, land cover, human host density, the availability of aquatic habitats for oviposition, and socio-economic variables among others [8–12]. Thus, small-scale entomological surveillance is likely required to predict human exposure risk at the local level.

In general, *Ae*. *aegypti* is highly associated with urban environments in tropical countries; where poor water and waste disposal infrastructure provide ample aquatic habitats for mosquito oviposition and larval development [13]. Although primarily urban, this species can also occur in peri-urban and rural areas [14–19]. The lower frequency of *Ae*. *aegypti* in peri-urban and rural settings may be due to the presence of other mosquito competitor species, and the reduced density of human hosts (given the high anthropophily of this species) and the artificial container habitats they prefer for oviposition [14,15,17–19]. Even within urban sites, *Ae*. *aegypti* can be highly heterogeneous [16,17,20]. Clusters often occur within 30 metres of households/neighbourhoods [21, 22]; matching the typical flight range of this species [23,24]. Understanding the drivers of local *Aedes* heterogeneity in urban centres is important for implementation of efficient surveillance and vector control. For example, as large-scale vector control across an entire city is time and resource consuming, focalized targeting at the neighbourhood or household level may be more cost effective [20,22,25,26].

Entomological surveillance can guide vector control programmes by directing efforts to areas and periods of highest vector abundance and/or human exposure [20,22,26]. *Aedes* surveillance systems measure presence and abundance in terms of larvae, pupae or adult mosquitoes [27,28]. However due to the complexity of arbovirus transmission, most of these entomological indicators are poorly correlated with disease incidence [28–30]. Of all mosquito life-history stages, the abundance of adult female *Aedes* (the only life stage capable of transmission) has shown the closest albeit weak association with DENV transmission [31]. However, more investigation is needed to identify which of the many methods available for adult *Aedes* surveillance is most indicative of human exposure risk [5,6,32].

Fine-scale analyses of *Aedes* vector behaviour and habitat use is also useful for selection of appropriate vector control. Control methods for adult *Aedes* often involve spraying of insecticides inside houses (Indoor Residual Spraying, IRS; [32]) or in outdoor peri-domestic areas (i.e., space spraying; [33]). The relative impact of such approaches will depend on both the susceptibility of vectors to insecticides, and the degree to which they rest inside houses versus outdoors. *Aedes aegypti* is considered to be highly endophilic [34]; however they can bite outdoors and rest in outdoor peri-domestic areas [35]. Assessment of the degree of outdoor resting and environmental determinants of this behaviour could thus be useful in planning *Aedes* control.

The coastal region of Ecuador experiences a higher incidence of DENV, CHIKV, and ZIKV than the rest of the country [36]. Vector control in this region typically consists of the application of temephos to larval habitats, IRS with deltamethrin and ultra-low volume (ULV) fogging with malathion to target the adult mosquitoes [37]. Arboviral transmission is generally higher during the rainy season (December to May) but fluctuates within and between urban areas likely in response to local environmental factors [38,39]. The primary aim of this study was to investigate the distribution, abundance, and resting behaviour (indoor versus outdoor) of adult *Ae*. *aegypti* populations at the neighbourhood level within two coastal cities of Ecuador. Additionally, we tested for associations between estimates of adult *Aedes* abundance made using different trapping methods and locations (BG sentinel traps, Prokopack indoor and peri-domestic aspirations) and weekly dengue incidence; with the aim of assessing which method was most indicative of epidemiological risk.

## Methods

### Ethics statement

Ethical approval was granted by the MVLS College Ethics Committee of the University of Glasgow (Project No.: 200150175), and by the Ethics Committee of Research on Human Beings of the San Francisco de Quito University (2016-146M).

### Research permits

A research permit was granted by the Ministry of Environment, Water and Ecological Transition (MAATE) to the San Francisco de Quito University was given through the Framework Agreement on Access to Genetic Resources No. MAE-DNB-CM-2016-0052. MAATE also granted permission to transport the samples to the University’s lab in Quito from Quinindé (MAE-DPAE-2017-0427-O) and from Portoviejo (MAE-CGZ4-DPAM-2017-0588-O).

### Study sites and period of study

**Fig 1:**
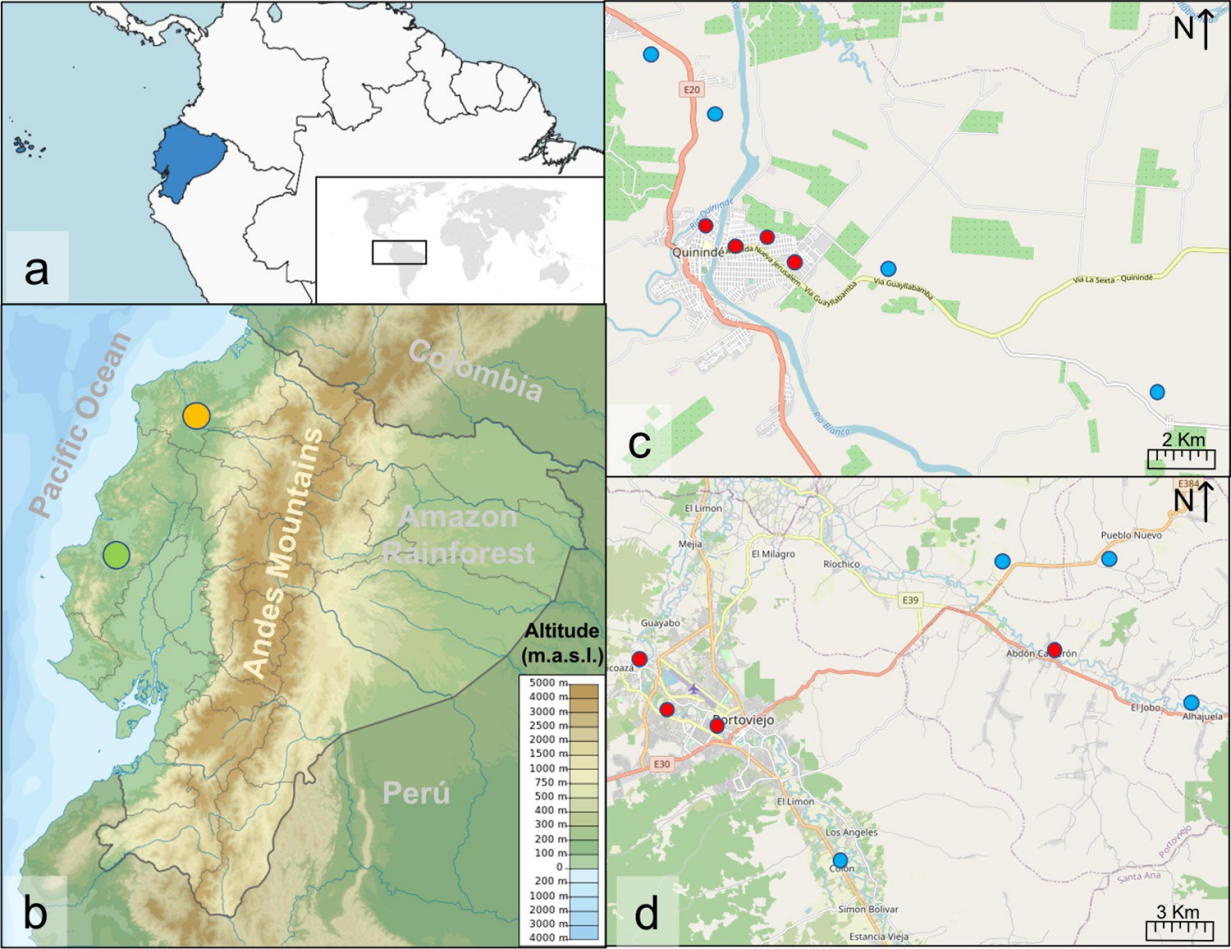
Map study sites. (a) Ecuador (highlighted in light blue) and its location in South America (South America background map layer was developed by ESRI, HERE, Garmin OpenStreetMap contributors and GIS user community [42], license public; Ecuador shapefile boundaries were taken from [43], whose licence is public domain; world map modified from [44], licence public domain); (b) location of the two cantons: Quinindé (orange circle) and Portoviejo (green circle) situated in the Pacific coastal region (taken from [45], license CC0 1.0 Universal Public Domain Dedication); (c) aerial view of Quinindé, with scale set at 2 km; and (d) aerial view of Portoviejo, with scale set at 3 km, with sampling points from urban (red dots) and peri-urban (blue dots) areas. Maps from panes (c) and (d) were taken from OpenStreetMap under the Open Data Commons Open Database License [46].

The study coincided with the tail end of a major ZIKV outbreak that occurred throughout the Americas in 2015–2017; capturing the arrival of ZIKV into Ecuador in late 2016/early 2017. It was carried out in two cantons in coastal Ecuador: Portoviejo (1.0°S, 80.4°W), province of Manabí, and Quinindé (0.3°N, 79.4°W), province of Esmeraldas (Fig 1A and 1B). In Ecuador, cantons are generally equivalent to cities and surrounding suburbs. The canton of Quinindé is approximately 3,875 km^2^, with an estimated population density of 36.3 people/km^2^ in 2017 [40, 41]. Mosquito collections were conducted in the urban parish (Quinindé-urban) of this canton (Fig 1C). Portoviejo canton including the city of Portoviejo is approximately 960 Km^2^ with an estimated population density of 326.63 people/km^2^ (2017). Mosquito collections were conducted in the urban parishes of Andrés de Vera, Colón, Picoazá, and Portoviejo; and the rural parishes of Abdón Calderón and Alhajuela.

Portoviejo sits at an altitude ranging between 30 and 150 m.a.s.l., while Quinindé ranges between 80 and 130 m.a.s.l. Both settings experience a wet, warm season running from December to May (average monthly rainfall of 1600mm, ~26°C mean daily temperature) and dry, cool season from June to November (average monthly rainfall of 450mm, ~24°C of mean daily temperature).

*Aedes* vectors were sampled at households within urban and peri-urban neighbourhoods in each canton. Neighbourhoods are not officially defined but take their definition and delimitations from a cultural perspective and historical belonging from the local communities [47]. Neighbourhoods categorized as “urban” were characterized by having households in a row-housing arrangement, usually organised in blocks surrounded by paved streets. Houses in urban neighbourhoods usually lacked open outdoor spaces like internal yards or gardens, and if present, these spaces were surrounded by walls. Neighbourhoods characterized as “peri-urban” were characterized by having fully detached houses scattered throughout the area, usually accessed by one main road. Wide lawn lots, crops and gardens surrounded peri-urban houses and properties were usually not limited by walls. Free movement of domestic animals between properties was possible in peri-urban neighbourhoods unless fences were present. Mosquitoes were sampled in each city over four periods between November 2016 and April 2017. Collection periods were approximately 45 days apart; with each consisting of three consecutive days of sampling. The first collection period in November fell within the dry, cooler season, with the others being the wet, warmer season.

In the last ten years, the two study sites have experienced a high incidence of arboviral diseases, with DENV occurring every year (Fig A in S1 Text). In 2016, both cantons experienced relatively similar DENV incidence (Portoviejo = 506.47 and Quinindé 514.31 cases per 100,000 population respectively), but in 2017 DENV incidence was considerable higher in Portoviejo than Quinindé (355.58 versus 61.85 cases per 100,000 population, respectively). Zika cases occurred in both study sites in 2016, with cases being much higher in Portoviejo than Quinindé (124.93 and 9.36 cases per 100,000 population, respectively (Fig A in S1 Text)).

### Trapping methods

Mosquito collections were carried out using methods that target different subgroups of adult *Aedes*: (1) BG-Sentinel (BGS) traps which target host seeking females (BioGents, Regensburg, Germany; Fig Ba in S1 Text); and (2) Prokopack aspirators that sample resting adults. BGS traps operate with a fan that pulls mosquitoes attracted by a lure into an internal collection bag [48]. BGS traps were baited with one BG-Lure cartridge and carbon dioxide (CO_2_)_._ Carbon dioxide was released by dry ice placed in a Coleman polyethylene cooler bottle (1.2 L capacity) placed inside traps. Baiting BGS with CO_2_ is known to increase their attractiveness to *Aedes* mosquitoes [49–51]. Prokopack (PPK) aspirators (John W. Hock, Gainesville, USA, [52]) were used to collect mosquitoes resting inside on house walls and ceilings, or in surrounding outdoor peri-domestic areas (Fig Bb in S1 Text). PPK are handheld aspirators that have an extendable 2-metre pole and an internal fan that pulls insects into a collection cup.

### Experimental design

At each site, four peri-urban and four urban neighbourhoods were selected for mosquito sampling (Fig 1C and 1D). Selection of the neighbourhoods was based on: (1) previously reported dengue cases, (2) presence of *Ae*. *aegypti* from previous government surveys, (3) informal recommendations from the local Ministry of Health personnel, (4) security and safety consideration and (5) access to logistics.

On each collection trip, mosquitoes were sampled from 24 households (three from each of eight neighbourhoods; in each canton). Different households from the same neighbourhoods were sampled on every collection trip; resulting in 12 households sampled in urban and peri-urban neighbourhoods on each trip (a total of 192 households sampled overall). On the first day of each collection period, houses were selected for sampling with assistance of a local guide; based on the availability of an outdoor area for peri-domestic sampling (garden/yard), residents reporting mosquito nuisance or recent arbovirus infection, and the willingness of residents to participate in the project (more information on the study design and selection of houses is in Fig C in S1 Text).

Within the three houses selected in each neighbourhood per field trip, one house was allocated for sampling with a BGS trap (daily collection for three consecutive days from the same house). BGS traps were set in the outdoor area and ran for an average of 9 hours daily (starting between 8–10 am, ending between 5–7 pm) to coincide with the known pattern of diurnal host seeking in *Ae*. *aegypti* [53]. Dry ice from the cooler bottles was replenished every morning. Mosquitoes were recovered from the traps at the end of each day with mosquitoes transferred into a plastic bag and placed in a cooler to kill them. Dead mosquitoes were transferred into 15 ml falcon tubes, pre-labelled with a unique code related to the sampling unit (trap type, date, trapping time, canton, neighbourhood type, neighbourhood name, and house ID) and stored in a cooler with dry ice for laboratory processing.

Sampling with Prokopack aspirators was conducted by two technicians for 10 minutes both inside and outside houses (between 10am and 5pm). One technician was randomly assigned to carry out aspiration indoors, and the other outdoors. Indoor aspirations were done by moving the aspirator nozzle along the walls, ceiling and under the furniture. Outdoor aspiration was carried out by aspirating along the outer sides of the walls, in external facilities like outdoor toilets, storage piles, garages, and laundry washing basins. Mosquitoes caught in Prokopack collection cups were transferred into 15 ml falcon tubes and placed in a cooler filled with dry ice to kill them.

### Mosquito processing and identification

Mosquito specimens were stored in dry ice and transported to the Medical Entomology and Tropical Medicine Laboratory (LEMMT) at Universidad San Francisco de Quito (USFQ) for processing. At LEMMT, specimens were counted, sexed and morphologically identified to species or genus level using taxonomical keys [54–56]. Identification of male specimens of *Cx*. *quinquefasciatus* was not done based on terminalia features as described by Bram (1967) [57], but rather assumed from the external features of the thorax, abdomen, head and legs using taxonomic keys [54,55].

### Environmental and epidemiological data

Microclimate data were recorded using TinyTag Plus 2 TGP-4500 (Gemini Co., UK) data loggers. Loggers were tied and hung inside each BGS trap during sampling. Measurements of air temperature and relative humidity were taken every 15 minutes and used to calculate the mean value per day. Macro climate data was obtained from the National Institute of Meteorology and Hydrology of Ecuador (INAMHI); including daily temperature and precipitation data from meteorological stations in Quinindé (M0156; 0.316°N, 79.469°W, 109 m.a.s.l.) and Portoviejo (M1208; 1.164°S, 80.390°W, 60 m.a.s.l.).

Weekly epidemiological data of dengue fever cases were obtained from SIVE-ALERTA government monitoring system from the Ministry of Health of Ecuador. The reported date corresponded to when the patient reported the onset of symptoms. Case reports were collected through passive surveillance procedures based on reporting at public and private health facilities (HF) in Portoviejo (71 HF) and in Quinindé (40 HF) [58]. Recorded cases of dengue were converted into incidence data by using population size of the given year from each canton as obtained from projections made by the National Institute of Statistics and Censuses of Ecuador (INEC) [41].

### Statistical analyses

Statistical analyses were carried out in R 3.5.0 and R Studio 1.1.419 using the packages “lme4”, “effects” and “multcomp” [59–61]. Generalized Linear Mixed Models (GLMM) and General Linear Hypotheses tests (GLHT–*Post Hoc* Tukey tests for GLMM) were used to assess variation in the abundance of *Ae*. *aegypti* females as a function of trap type (BGS, Prokopack-IN, Prokopack-OUT), location (Portoviejo or Quinindé), neighbourhood type (urban or peri-urban), month of collection (November 2016, January 2017, March 2017 and April 2017), and mean daily temperature (taken from INAMHI’s weather stations). As mosquito abundance data were highly overdispersed, models were fitted with a negative binomial distribution [62]. Additionally, estimates of cumulative past rainfall were calculated (using data from INAMHI weather stations) and incorporated into models as a proxy of water availability during the larval development of *Ae*. *aegypti* adults caught in traps. Based on the known *Ae*. *aegypti* life cycle (Christophers 1960 [53]), adult females caught in a trap would have arisen from eggs laid ~16–39 days previously (Fig D in S1 Text); consequently the cumulative rainfall in the study area 3, 2 weeks and 1 week before collection were incorporated into models of adult abundance. Models also tested for interactions between month of collection and location, neighbourhood type and location, trapping method and location, neighbourhood type and trapping method, and mean daily temperature and location. Collection date, neighbourhood, house ID, and trap and collector ID were included as random effects. Initially, two model structures were built which differed in regard to how rainfall was included (1) the first model included all of the described explanatory variables, plus rainfall included at three different lags (Table A in S1 Text, Model 1). The second model structure was similar except that rainfall was included as only one covariate, representing the cumulative rainfall over the three-week period before mosquito collection (Table A in S1 Text, Model 2). Maximal models from both model structures were compared using the Akaike Information Criterion (AIC) [63]; with the model with the lowest AIC retained for further model selection to assess the significance of covariates through backward stepwise elimination using Likelihood Ratio Tests (LRT) [64].

To test the impact of microclimate (as measured at the trapping station) on the host seeking behaviour of *Ae*. *aegypti*, further analysis was conducted just on the subset of data from BGS traps where temperature and humidity were measured by TinyTag loggers (used in 3 out of 4 collection trips, in January–April 2017). Here the response variable was the daily abundance of female *Ae*. *aegypti*, with the explanatory fixed and random variables used being the same as in the analysis of the full dataset except that the measure of mean daily temperature and humidity was obtained from dataloggers at the household rather than from weather stations. Additionally, interactions between temperature and humidity, temperature and location, and humidity and location were also included as fixed effects (Table A in S1 Text, Model 3).

Finally, in order to test for a link between entomological and epidemiological data, weekly dengue fever incidence and mean *Aedes* abundance were estimated for that same week, and one and two weeks before the DENV cases were reported. Weekly data was standardized according to the corresponding epidemiological week (EW) defined by the World Health Organization [65]. First, mean weekly estimates of female *Ae*. *aegypti* abundance were obtained from collections made in Portoviejo and Quinindé using GLMMs as described above. Here, female *Aedes* abundance was modelled as a function of EW of collection, canton, and mosquito trapping method (all fixed effects), with collection day, household ID and trap ID included as random effects. In scenarios where the 3 consecutive days of mosquito collections did not fall in the same epidemiological week, the assigned EW was that corresponding to two of the three days of sampling. The response variable was fit to a negative binomial distribution to account for overdispersion. Three separate Generalized Linear Models (GLM) were used to model dengue fever incidence as a function of female *Aedes* abundance during the same week of collection, and one and two weeks after the entomological surveillance took place. All figures were created using the packages “ggplot2” and “ggpubr” [66,67].

## Results

A total of 3987 mosquitoes from 7 different genera were collected during the study. For full details of species and numbers caught, see Tables B and C in S1 Text.

### Abundance and behaviour of *Ae*. *aegypti*

Of two alternative model structures compared for analysing variation in *Ae*. *aegypti* abundance (differing in how lagged rainfall effects were considered), neither had a clear advantage over the other (difference in AIC between the models was smaller than 2 units; Δ = 0.096) [63]. However, the model that incorporated rainfall as three separate time-lagged covariates had a slightly lower AIC and therefore was retained for further analysis and evaluation of covariates (Table A in S1 Text, Model 1).

**Fig 2:**
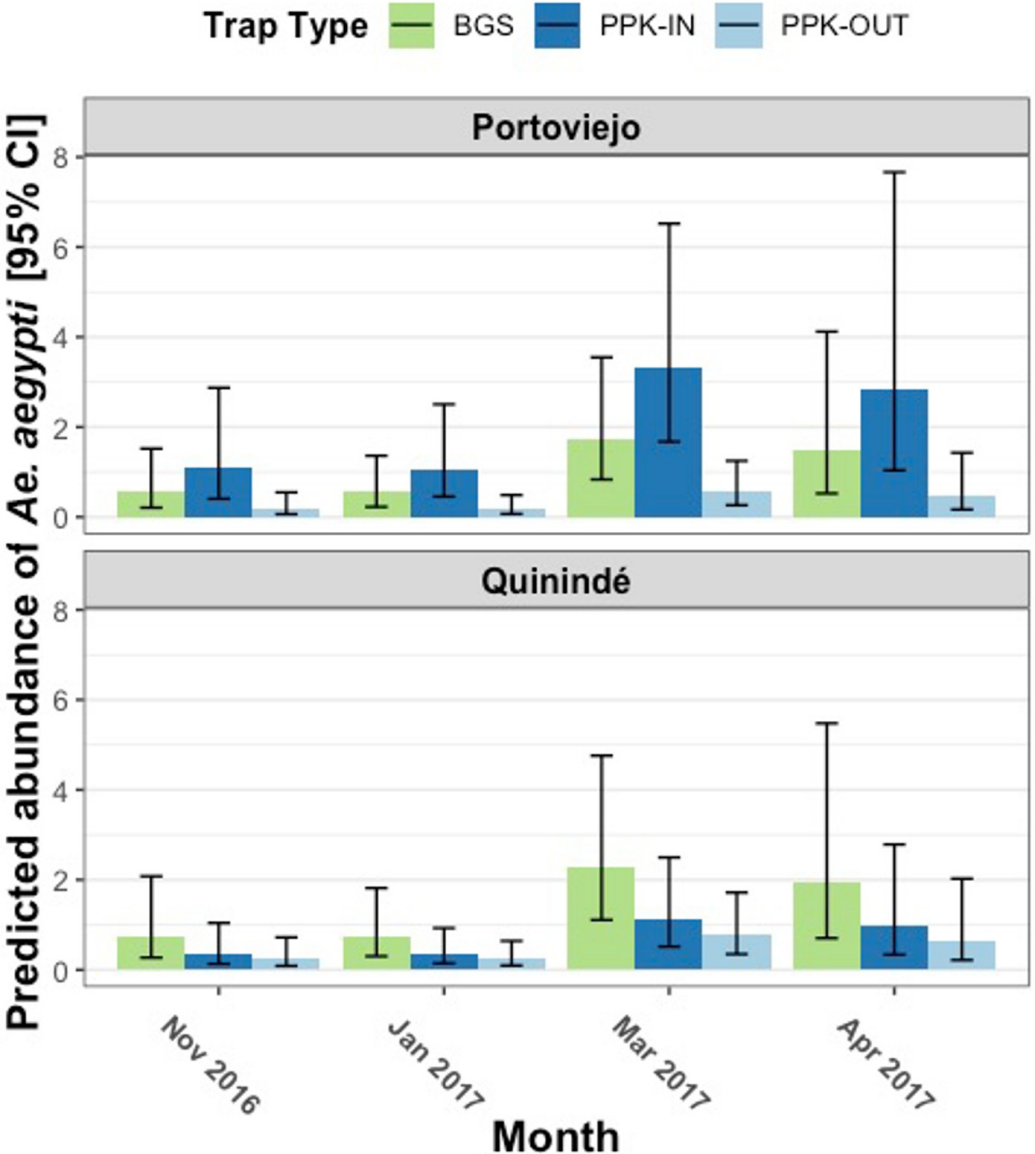
Predicted *Ae*. *aegypti* female abundance according to month of collection per canton. Height of columns indicate the estimated mean of *Ae*. *aegypti* females per trapping collection, while error bars indicate the 95% CI. Different colours of bar represent different trapping methods, being BG-Sentinel trap (BGS), Prokopack aspirations made inside (PPK-IN) or outside of houses (PPK-OUT).

Based on this model, *Ae*. *aegypti* abundance was significantly associated with the month of collection (*X*^*2*^ = 10.11, df = 3, *p* = 0.02), cumulative rainfall 3 weeks prior to collection (28–22 days prior; *X*^*2*^ = 5.07, df = 1, *p* = 0.02), neighbourhood type (*X*^*2*^ = 8.60, df = 1, *p*<0.01), and the interaction between location (canton) and trap type (*X*^*2*^ = 19.83, df = 2, *p*<0.001; Table D in S1 Text). *Ae*. *aegypti* female abundance was significantly higher in March 2017 (wet, warm season) than in November 2016 (cool, dry season; Fig 2 and Table E in S1 Text, GLHT Tukey: Z = 2.56, *p* = 0.04) and January 2017 (transition between cool, dry and wet, warm season; Fig 2 and Table E in S1 Text, GLHT Tukey: Z = 2.88, *p* = 0.02). There was no difference in mean abundance between months of collections in the rest of the pairwise combinations.

**Fig 3:**
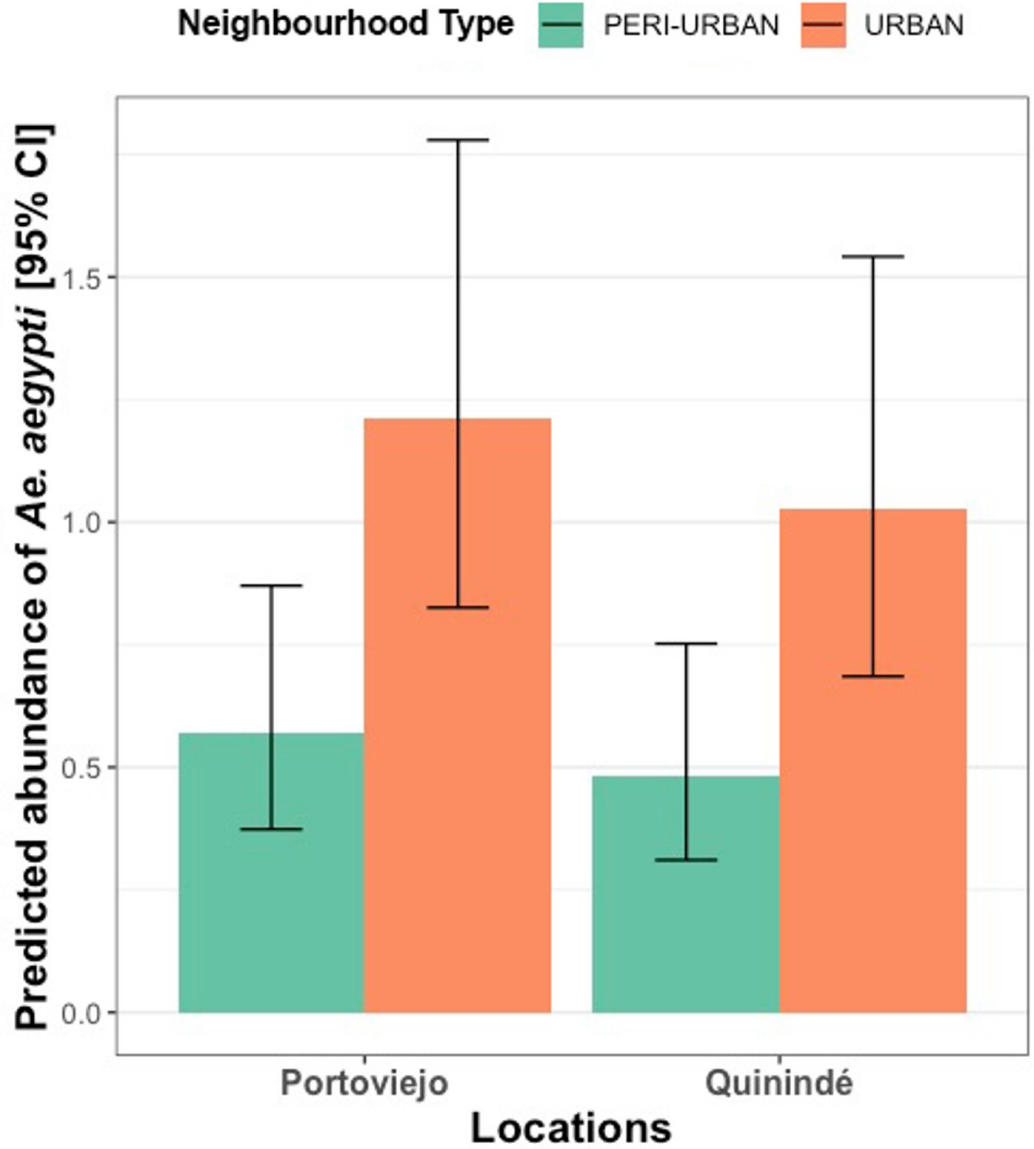
Predicted *Ae*. *aegypti* female abundance according to neighbourhood type per canton. Height of columns indicate the estimated mean of *Ae*. *aegypti* females, while error bars indicate the 95% CI. Different colours of bar represent a different neighbourhood type.

The abundance of female *Ae*. *aegypti* was approximately two times higher at households in urban than peri-urban neighbourhoods (*X*^2^ = 8.6, df = 1, *p*< 0.01, Fig 3 and Tables D and E in S1 Text).

**Fig 4:**
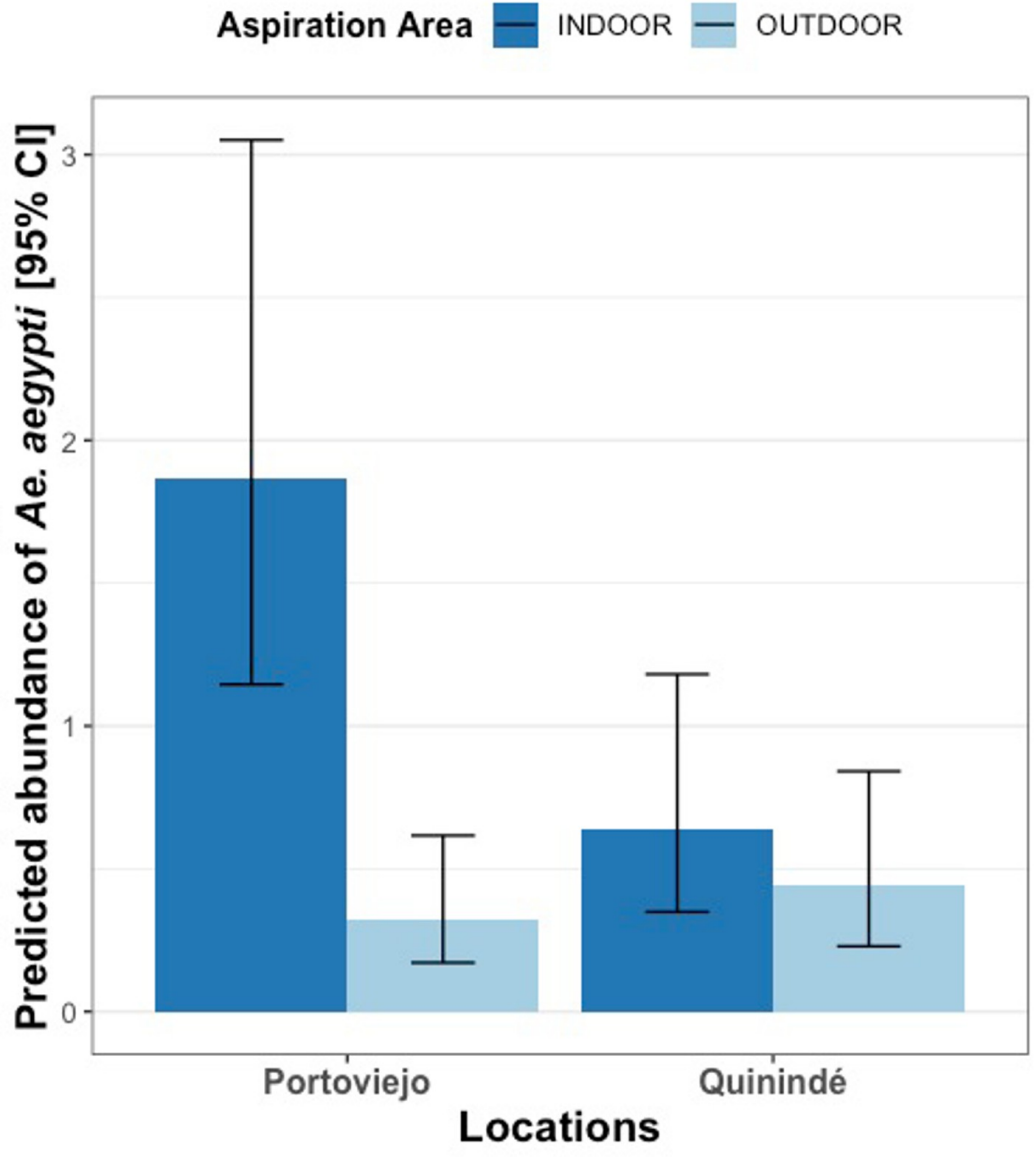
Predicted *Ae*. *aegypti* female abundance in indoor or outdoor Prokopack aspiration collections, in different cities. Height of columns indicate the estimated mean of *Ae*. *aegypti* females per collection, while the error bars indicate the 95% CI. Different colours of bar represent whether mosquitoes were collected in Prokopack aspiration made inside or outside of houses.

There was no consistent difference in *Ae*. *aegypti* abundance between cities; with the relative difference depending on the mosquito trapping method used (Table D in S1 Text). In collections made with indoor Prokopack aspirations, *Ae*. *aegypti* females were three times more abundant in Portoviejo than Quinindé (Fig 4 and Table E in S1 Text, GLHT Tukey: Z = -3.56, *p*<0.01), but there was no difference between cities in the number caught in outdoor aspirations (Fig 4 and Table E in S1 Text, GLHT Tukey: Z = 0.87, *p* = 0.95). In Portoviejo, *Ae*. *aegypti* females were 6-fold higher in indoor versus outdoor Prokopack aspirations (Fig 4 and Table E in S1 Text, GLHT Tukey: Z = -6.73, *p*<0.001), with no difference between outdoor and indoor collections in Quinindé (Fig 4 and Table E in S1 Text, GLHT Tukey: Z = -1.40, *p* = 0.72). The abundance of *Ae*. *aegypti* females in BGS traps was also similar in Portoviejo and Quinindé (Fig 2 and Table E in S1 Text, GLHT Tukey: Z = 0.91, *p* = 0.94). BGS traps collected significantly more female *Ae*. *aegypti* than outdoor Prokopack aspirations in both cities (Fig 2 and Table E in S1 Text, **Portoviejo:** GLHT Tukey: Z = -3.90, *p*< 0.01; **Quinindé:** GLHT Tukey: Z = -4.07, *p*< 0.001); but were not significantly different from indoor Prokopack aspirations (Fig 2 and Table E in S1 Text, **Portoviejo:** GLHT Tukey: Z = 2.72, *p* = 0.07; **Quinindé:** GLHT Tukey: Z = -2.76, *p* = 0.06).

**Fig 5:**
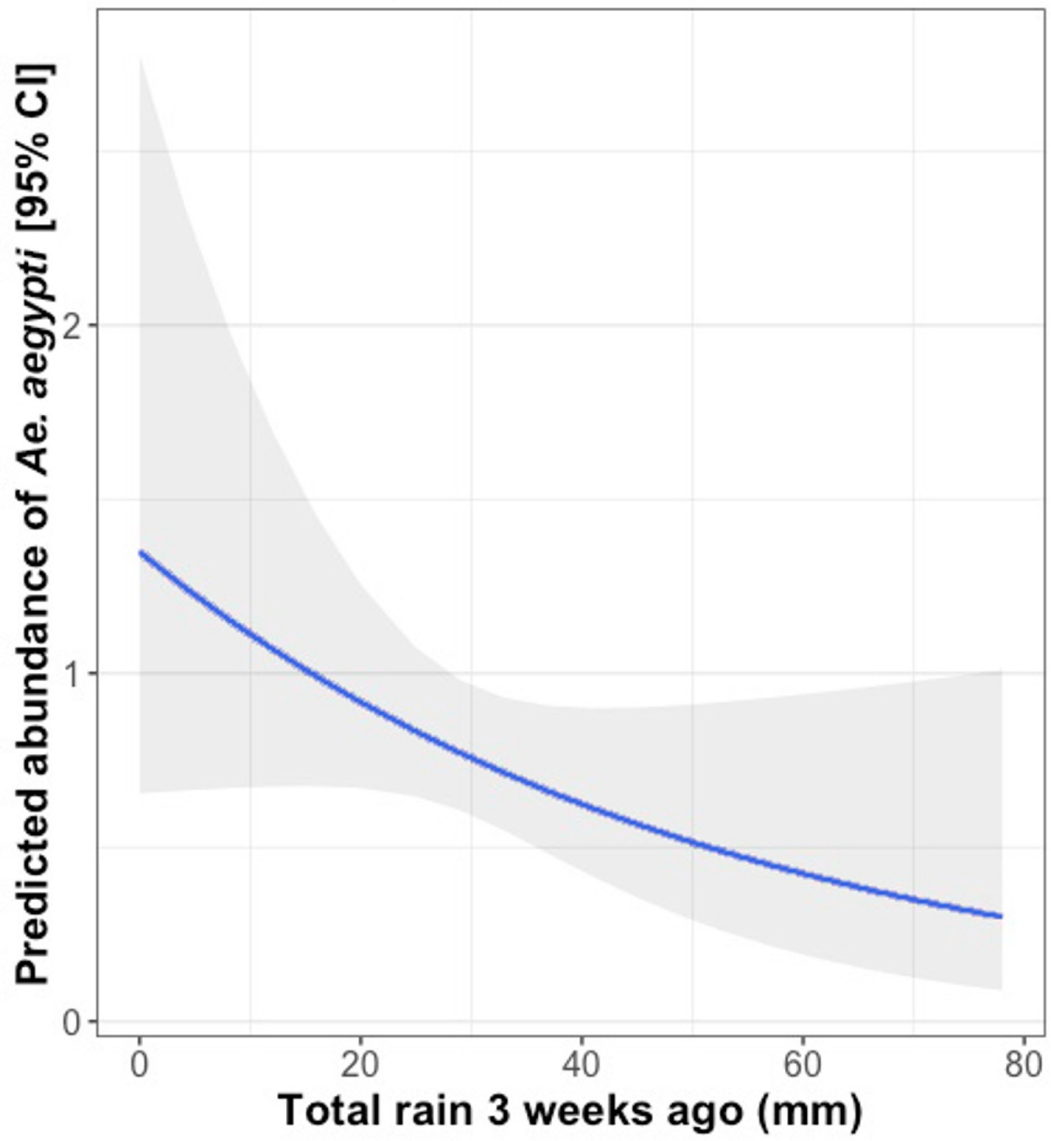
Predicted association between *Ae*. *aegypti* female abundance according and the volume of rainfall falling 28 to 22 days before collection day. The blue line indicates the estimated mean of *Ae*. *aegypti* females, while the grey shaded area indicates the 95% CI.

Finally, there was a significant negative association between the cumulative rainfall occurring in the third week before collection (28 to 22 days prior) and the mean daily abundance of *Ae*. *aegypti* (Fig 5 and Table D in S1 Text).

Restricting analysis to the data set for which precise microclimatic measurements at trapping points were available (BGS collections, January to April 2017), the abundance of *Ae*. *aegypti* females also varied between collection months (*X*^*2*^ = 12.84, df = 2, *p*<0.01) by being higher towards April, and was similarly predicted to decrease in relation to rainfall (*X*^*2*^ = 8.68, df = 1, *p*<0.01) occurring 22–28 days before collections. However, there was no significant impact of local temperature or humidity at the trapping point (Table F in S1 Text).

### Association between *Aedes aegypti* abundance and dengue incidence

Dengue transmission occurred throughout both years of the study in each canton (Fig A in S1 Text). Incidence was high in both cantons in 2016, but with a pronounced decline in the second half of the year in Quinindé compared to Portoviejo. In 2017, transmission remained high during the warm, wet season (Jan-July) in Portoviejo. DENV incidence in Quinindé was much lower in 2017 than the previous year, and in comparison to Portoviejo.

**Fig 6:**
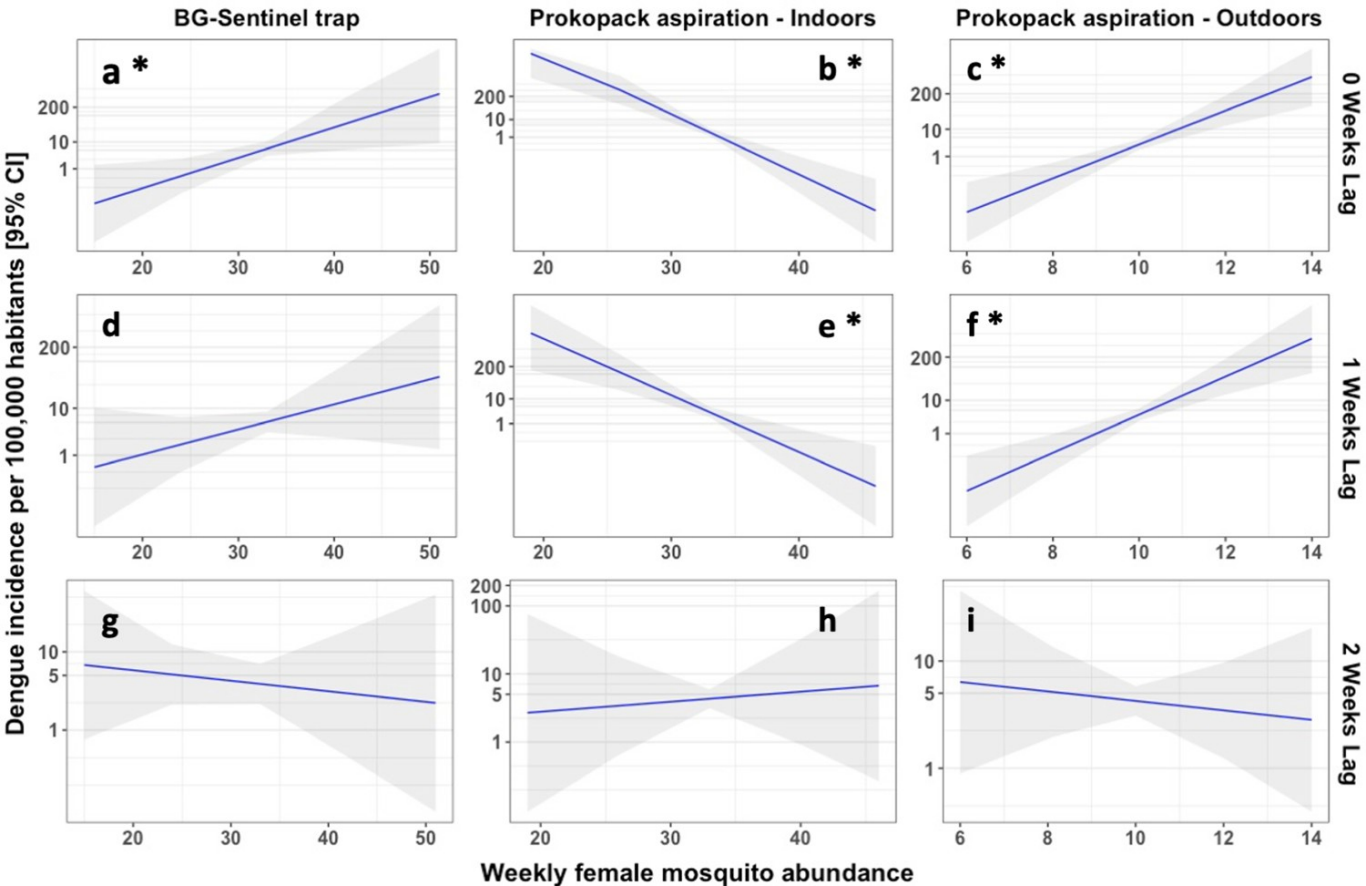
Effect of female *Aedes* abundance on dengue fever incidence during 3 lag periods. Predicted mean incidence of dengue fever in Portoviejo and Quinindé during 2016 and 2017 given by female *Aedes aegypti* abundance. Columns represent the trapping method used to collect *Aedes* female mosquitoes, and rows represent the lag periods. Asterisks (*) next to the pane label indicate significant relationships. The trend of the relationship is represented by the solid blue line and shaded areas around the blue lines indicate the 95% confidence intervals.

Using these data, we tested for associations between mean *Ae*. *aegypti* abundance and DENV incidence on the same week of collections, and with *Ae*. *aegypti* abundance one and two weeks before the dengue reported case. These analyses generated contradictory results. Estimates of *Ae*. *aegypti* abundance derived from BGS (on same week) and outdoor PPK (on same week and one week before) were positively correlated with incidence, however abundance estimates from indoor PPK collections (on same week and one week before) were negatively correlated with incidence (Fig 6 and Table G in S1 Text).

## Discussion

Surveillance of *Ae*. *aegypti* vector populations was conducted in two urban hotspots of dengue transmission in coastal Ecuador with the aim of investigating drivers of local heterogeneity in abundance and resting behaviour. There was considerable heterogeneity in *Ae*. *aegypti* abundance and resting behaviour within the two study sites. *Aedes aegypti* were more abundant during the wet and warm months (March and April) than in cooler months of November and January. There was also substantial variation in *Ae*. *aegypti* abundance between neighbourhoods; with vectors being two times more abundant in urban than in peri-urban neighbourhoods. There was no consistent difference in vector abundance between cities due to an interaction between study site and trapping method. Specifically, while *Ae*. *aegypti* was much more abundant in indoor than peri-domestic resting collections in Portoviejo, this vector was similarly abundant in indoor and outdoor resting collections in Quinindé. Associations between mean *Ae*. *aegypti* abundance and weekly dengue incidence were varied and differed depending on the mosquito trapping method used. These results highlight the considerable heterogeneity in *Ae*. *aegypti* ecology and its predicted association with epidemiological risk in coastal Ecuador.

*Aedes aegypti* were most abundant in the warm and wet months (March and April), coinciding with the highest DENV transmission. This matches observations from the southern coast of Ecuador [68], and other countries where *Ae*. *aegypti* abundance peaks during the rainy and warm season [69–71]. Implementation of proactive vector control during the months before the *Ae*. *aegypti* peak may be most effective in these settings [1,4]. More extensive year-round vector surveillance over multiple years is recommended to confirm the repeatability of *Aedes* seasonality in these settings in coastal Ecuador, and plan vector control accordingly.

Climate is known to be an important driver of *Ae*. *aegypti* population dynamics [72,73]. In contrast to previous studies, here we found no association between mean daily *Ae*. *aegypti* abundance and temperature and humidity as recorded at nearby weather stations. This lack of association could be due to the relatively low variation in these environmental variables over the sampling months. The only micro-climatic variable associated with *Aedes* abundance was cumulative rainfall occurring 22–28 days before sampling (negative association). The mechanism behind this negative association is unclear, but may be due to increased water storage during periods of low rain that creates more aquatic habitats for oviposition and larval developments [74].

As expected, *Ae*. *aegypti* females were also more abundant in urban than in peri-urban neighbourhoods. The ability of *Ae*. *aegyp*ti to adapt to urban environments is well known [15,75], with lower abundance in peri-urban areas likely due to the reduced availability of artificial container habitats for larvae [11,76]. Heterogeneity of living standards between urban and peri-urban neighbourhoods may also account for the variation in *Ae*. *aegypti* reported here. For instance, in these cities, households in urban neighbourhoods are often smaller and more economically-disadvantaged than those in peri-urban settings. Households in urban neighbourhoods had lower access to piped water resulting in a greater need to store water in large containers around homes, thus creating larval habitats [74–76]. Limited access to infrastructure in these neighbourhoods is further compounded by high population densities, providing ample blood feeding sources for *Aedes* females [34, 77–80]. Preferentially, targeting vector control to these high-density, economically-disadvantaged neighbourhoods may be more cost effective than a city-wide vector control approach [81]. However, *Aedes* vectors were also consistently found in peri-urban neighbourhoods, indicating these areas should not be ignored in surveillance and control activities.

Aspiration using Prokopack [41] or other methods [82] are highly efficient for sampling *Ae*. *aegypti* in urban areas; with densities typically being considerably higher in collections made inside houses than surrounding outdoor peri-domestic areas [32,34,83,84]. This observation was repeated in Portoviejo where estimated abundance of *Ae*. *aegypti* females was six times higher in aspirations made inside than outside, but not Quinindé where abundance was relatively similar in indoor and outdoor collections. These canton-level differences might be due to a combination of factors including genetic variation in *Ae*. *aegypti* populations, their resistance to insecticides used indoors and others [85]. The notable difference in endophily between these two *Ae*. *aegypti* populations highlights the importance of studying local vector ecology as epidemiologically-relevant behaviours can vary even between similar urban settings.

Although *Ae*. *aegypti* abundance is frequently assumed to be a proxy for DENV transmission (e.g., [86]), most vector indices are poor predictors of arboviral incidence [28]. The lack of concordance between vector density and human infection risk may be due to biases in *Aedes* sampling methods [29,85] which capture the abundance of different life stages but not direct biting rates on humans. Given the high expense and logistics involved with epidemiological monitoring in human populations, there would be great value in finding appropriate entomological indicators of risk. However, results from this study indicate that the association between *Ae*. *aegypti* abundance and dengue fever incidence differed among trapping methods. Weekly DENV incidence was positively associated with *Ae*. *aegypti* abundance as estimated from BGS traps and outdoor PPK aspirations made during the same week (and with counts from outdoor PPK aspirations made 1 week previously). However, DENV incidence was negatively correlated with abundance estimates derived from PPK collections made inside houses (both concurrently week or one week before dengue reporting). Despite the inconsistency of these associations, our results indicate the presence of predictive associations between *Aedes* abundance (trap-dependent) with arbovirus incidence [29]. Given the entomological (12 households/week) and epidemiological (city-wide) data were not collected at the same spatial scale, this evidence of even some statistical association is encouraging with respect to the use of entomological indicators of transmission. We encourage further investigation of the use of vector abundance data using different trapping methods, including in peri-domestic as well as indoor settings, to predict DENV incidence trends. Ideally future studies should also include finer-scale epidemiological data to facilitate geostatistical analyses of entomological and epidemiological variables over smaller and concurrent spatial scales.

An important limitation in our approach of testing entomological indicators of epidemiological risk was the lack of information on the infection status of mosquitoes. Dengue positivity in mosquito populations can be a strong predictor of the timing of local outbreaks [87]. Therefore, routine testing for arboviral presence would be useful to incorporate in future studies, ideally using low cost and simple infection detection methods such as the one used by Lau *et al*. [87,88]. Additionally, this study has a number of further limitations that require investigation. First, although this study was concentrated on the highest transmission period of the year (encompassing the rainy season of a ZIKV epidemic year, with active transmission of CHIKV and DENV), further surveillance into the dry season would be required to fully characterize seasonal dynamics and capture the extremes of environmental conditions which may impact *Ae*. *aegypti* populations. Year-round surveillance of *Ae*. *aegypti* populations over multiple years would be of great value. Longer-term surveillance would also permit more robust analysis of micro and macro climatic drivers of vector populations and DENV transmission. Furthermore, future studies would benefit from concurrent entomological and epidemiological surveillance (human case incidence) across the year, to provide a stronger foundation for assessment of the potential impact of vector control on human infection and disease. Finally, it would be useful to incorporate more detailed data on vector control practices at the neighbourhood and household level and how they relate to differences between *Aedes* vector populations.

In conclusion, our findings confirm that *Ae*. *aegypti* abundance is highly heterogeneous at the household and neighbourhood level and reveal unexpected heterogeneity in endophily; with differential use of indoor and peri-domestic resting habitats by *Aedes* at two urban sites. This behavioural variation highlights that applying vector control/spraying to cover peri-domestic areas could add considerable value in some but not all areas. In combination, these results identify the potential value of implementing tailored vector control activities based on the local ecological and environmental conditions.

## Supporting information

S1 Text Fig A. Weekly reported dengue and Zika virus incidence estimated from cases reported in 2016 and 2017. Incidence of dengue and Zika is shown for Quinindé and Portoviejo during 2016 and 2017. Fig B. Trapping methods used in this study. (a) Typical set up of a BGS trap. (b) Technician using a Prokopack aspirator for outdoor sampling. Photographs taken by author LDOL. Fig C. Experimental design. Schematic diagram of the experimental design used to sample mosquitoes from two cantons in Ecuador, Portoviejo and Quinindé, across 4 collection periods: November 2016, January, March and April 2017. The study took place in 4 urban and 4 peri-urban neighbourhoods at each canton. Three households (H1, H2, and H3) were sampled from each neighbourhood with different houses sampled on each of the 4 collection periods, giving a total of 12 households per neighbourhood over all 4 sampling trips). Source: see Acknowledgments section. Fig D. Time of development of Ae. aegypti females along their life stages. Development time of *Ae*. *aegypti* females according to Christophers 1960 [42]. The duration time from when eggs have been oviposited (A) to the first larval instar (B), pupal stage (C), a newly emerged adult female (D), a female that hastaken her first blood meal (E), and when that female will oviposit eggs produced from that first blood meal (F). Source: see Acknowledgments section. Table A. Full model structures. Three model structures for statistical analyses were tested in this study and full models are shown. Table B. Abundance of mosquitoes collected with BG-Sentinel (BGS) traps and Prokopack (PPK) aspirations in Portoviejo and Quinindé between November 2016 and April 2017. Mosquitoes are broken down by sex (♂ = males, ♀ = females), with females further split by blood feeding status. PPK aspirations were carried out inside houses and in the outdoor area for 10 minutes at each house area, while BGS collections were carried out outdoors for approximately 9 hours during the day. Table C. Abundance of mosquitoes collected with BG-Sentinel (BGS) traps and Prokopack (PPK) aspirations in Portoviejo and Quinindé between November 2016 and April 2017. Mosquito species are broken down by period of collection and trapping method. PPK aspirations were carried out inside houses and in the outdoor area for 10 minutes at each house area, while BGS collections were carried out outdoors for approximately 9 hours during the day. Table D. Summary table of statistical significance of explanatory variables tested for association with *Ae*. *aegypti* female abundance. Significance values for each of the explanatory variables from the fitted models. Values of chi-square (*X*^*2*^), degrees of freedom (df), and *p*-values for each of the covariates tested are shown. Bold values with an asterisk (*) indicate significant terms. Fixed effects with a double S symbol (§) indicate the interaction term. “NA” indicates “not applicable” values for which single term significance was not possible because of their involvement in significant interaction terms. The letter “w” means week. Table E. Estimated mean abundance of *Ae*. *aegypti* females. Mean values are given for each month of collection neighbourhood type, and canton and trap type combination, with the corresponding 95% CI of the lower and upper limits. Values for each of the three trapping methods, BG-Sentinel traps (BGS) and indoor Prokopack aspirations (PPK-IN) and outdoor (PPK-OUT) are given too. Table F. Summary table of significance of variables tested for microclimatic association with Ae. aegypti female abundance. Significance values for each of the explanatory variables from the fitted models. Values of chi-square (*X*^*2*^), degrees of freedom (df), and *p*-values for each of the covariates tested are shown. Bold values with an asterisk (*) indicate significant terms. Fixed effects with a double S symbol (§) indicate the interaction term. “NA” indicates “not applicable” values for which single term significance was not possible because of their involvement in significant interaction terms. The letter “w” means week. Table G. Summary table of statistical significance of explanatory variables tested for association with dengue incidence. Analysis based on a subset of incidence data corresponding to the timing of *Aedes* vector surveillance carried out in each canton between November 2016 and April 2017. Values of chi-square (*X*^*2*^), degrees of freedom (df), and *p*-values for each of the predictors tested are shown. Bold values with an asterisk (*) indicate significant terms. “NA” indicates “not applicable” values for which single term significance was not possible because of their involvement in significant interaction terms.
(DOCX)
