# Supplementary material for: Behaviour and distribution of Aedes aegypti mosquitoes and their relation to dengue incidence in two transmission hotspots in coastal Ecuador
Source: PLoS Negl Trop Dis. 2024 Apr 29;18(4):e0010932. doi: 10.1371/journal.pntd.0010932 (PMC11081501; doi:10.1371/journal.pntd.0010932)
Supplement: S1 Text — Fig A. Weekly reported dengue and Zika virus incidence estimated from cases reported in 2016 and 2017. Incidence of dengue and Zika is shown for Quinindé and Portoviejo during 2016 and 2017. Fig B. Trapping methods used in this study. (a) Typical set up of a BGS trap. (b) Technician using a Prokopack aspirator for outdoor sampling. Photographs taken by author LDOL. Fig C. Experimental design. Schematic diagram of the experimental design used to sample mosquitoes from two cantons in Ecuador, Portoviejo and Quinindé, across 4 collection periods: November 2016, January, March and April 2017. The study took place in 4 urban and 4 peri-urban neighbourhoods at each canton. Three households (H1, H2, and H3) were sampled from each neighbourhood with different houses sampled on each of the 4 collection periods, giving a total of 12 households per neighbourhood over all 4 sampling trips). Source: see Acknowledgments section. Fig D. Time of development of Ae. aegypti females along their life stages. Development time of Ae. aegypti females according to Christophers 1960 [42]. The duration time from when eggs have been oviposited (A) to the first larval instar (B), pupal stage (C), a newly emerged adult female (D), a female that hastaken her first blood meal (E), and when that female will oviposit eggs produced from that first blood meal (F). Source: see Acknowledgments section. Table A. Full model structures. Three model structures for statistical analyses were tested in this study and full models are shown. Table B. Abundance of mosquitoes collected with BG-Sentinel (BGS) traps and Prokopack (PPK) aspirations in Portoviejo and Quinindé between November 2016 and April 2017. Mosquitoes are broken down by sex (♂ = males, ♀ = females), with females further split by blood feeding status. PPK aspirations were carried out inside houses and in the outdoor area for 10 minutes at each house area, while BGS collections were carried out outdoors for approximately 9 hours during the day. T [file pntd.0010932.s001.docx]

**S1 Text**

**METHODS**


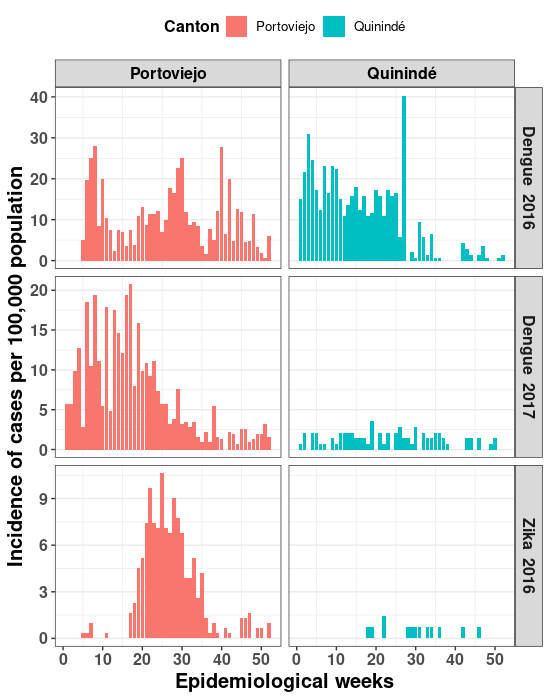


**Fig A. Weekly reported dengue and Zika virus incidence estimated from cases reported in 2016 and 2017.** Incidence of dengue and Zika is shown for Quinindé and Portoviejo during 2016 and 2017.


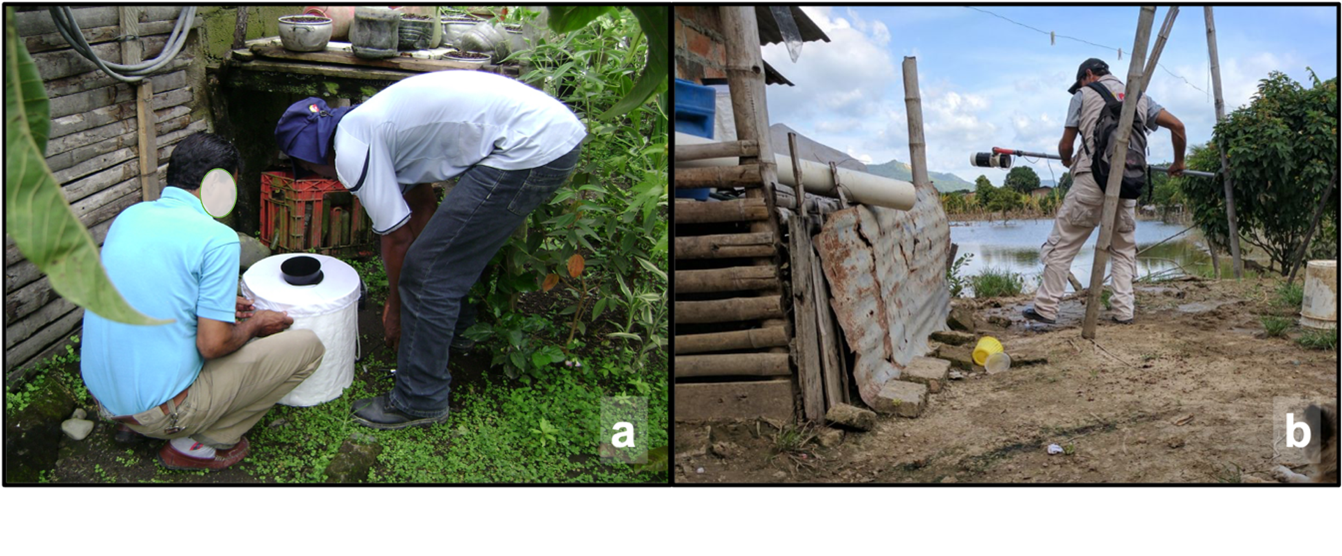


**Fig B***.* **Trapping methods used in this study.** (a) Typical set up of a BGS trap. (b) Technician using a Prokopack aspirator for outdoor sampling. Photographs taken by author LDOL.


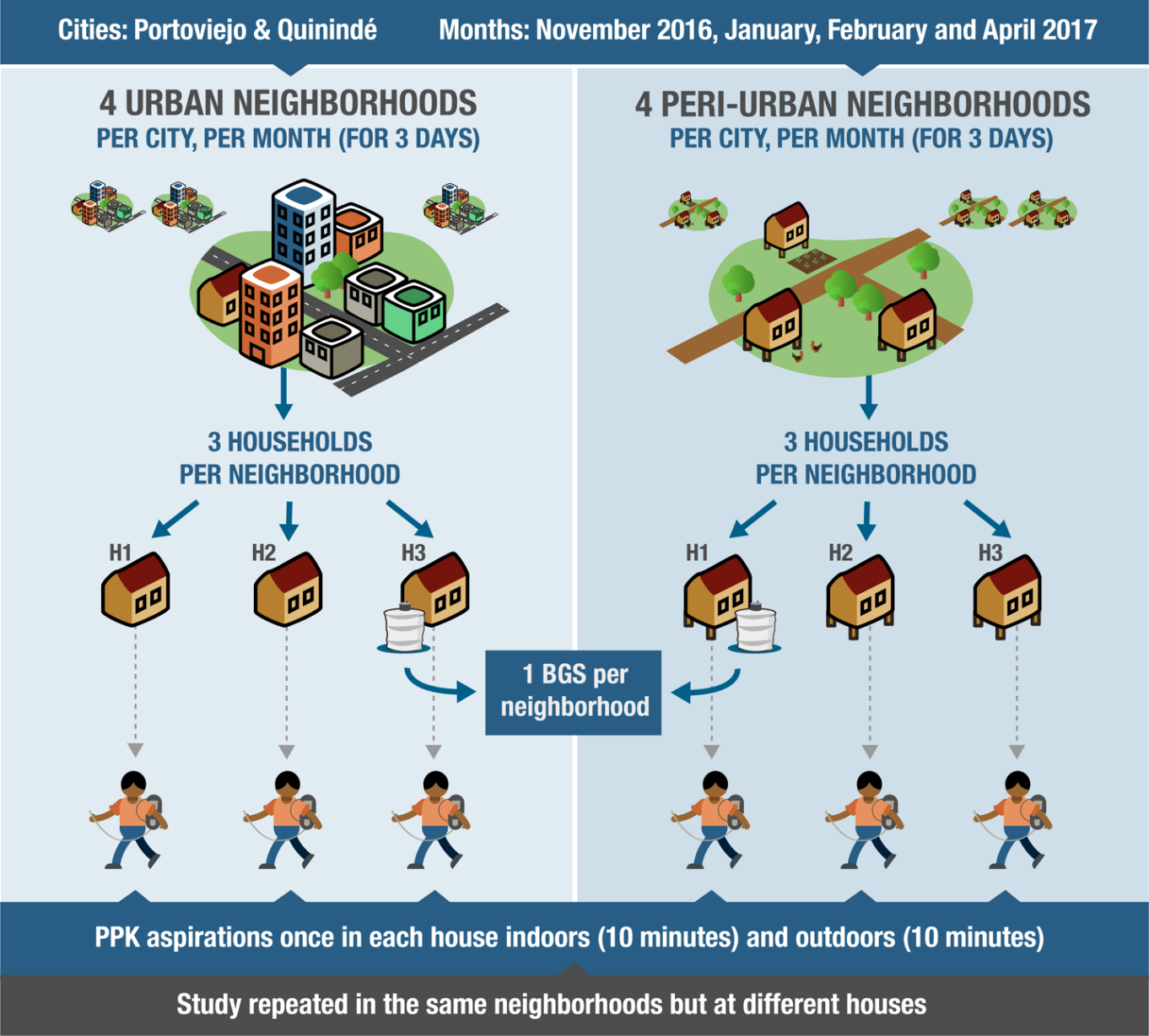


**Fig C**. **Experimental design**. Schematic diagram of the experimental design used to sample mosquitoes from two cantons in Ecuador, Portoviejo and Quinindé, across 4 collection periods: November 2016, January, March and April 2017. The study took place in 4 urban and 4 peri-urban neighbourhoods at each canton. Three households (H1, H2, and H3) were sampled from each neighbourhood with different houses sampled on each of the 4 collection periods, giving a total of 12 households per neighbourhood over all 4 sampling trips).


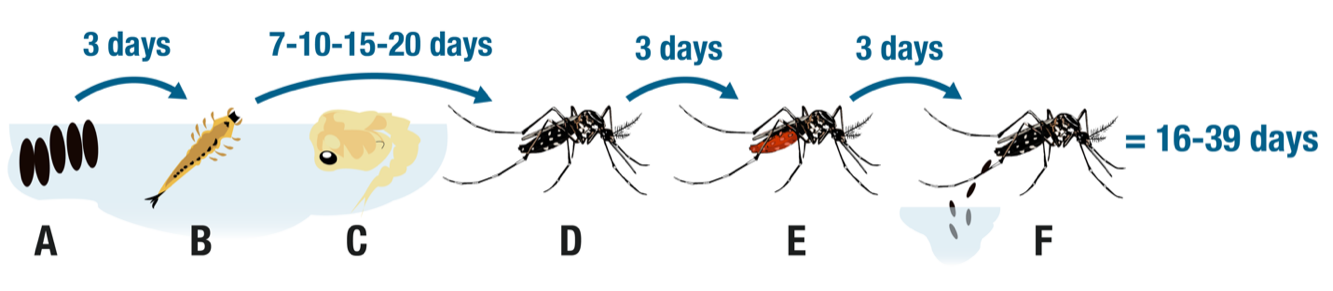


**Fig D. Time of development of *Ae. aegypti* females along their life stages.** Development time of *Ae. aegypti* females according to Christophers 1960 [42]. The duration time from when eggs have been oviposited (A) to the first larval instar (B), pupal stage (C), a newly emerged adult female (D), a female that has taken her first blood meal (E), and when that female will oviposit eggs produced from that first blood meal (F).

**Table A. Full model structures.** Three model structures for statistical analyses were tested in this study and full models are shown.

| **Model**  **structure** | **Response**  **variable** | **Fixed effects** | **Random effects** |
| --- | --- | --- | --- |
| 1 | AeAb | Mo + Loc + Nt + Tr + Tmp + Ra_1_ + Ra_2_ + Ra_3_ + Loc*Nt + Loc*Tr + Loc*Mo + Nt*Tr + Tmp*Loc | Dt + Nh + Hs + Col |
| 2 | AeAb | Mo + Loc + Nt + Tr + Tmp + Ra_1,2,3_ + Loc*Nt + Loc*Tr + Loc*Mo + Nt*Tr + Tmp*Loc | Dt + Nh + Hs + Col |
| 3 | AeAb_1_ | Mo + Loc + Nt + Tmp_1_ + Hum + Ra_1_ + Ra_2_ + Ra_3_ + Loc*Mo + Tmp_1_*Hum + Tmp_1_*Loc + Hum*Loc | Dt + Nh + Hs + Col |
|  |  |  |  |
| **Abbreviation** | | **Description** | |
| AeAb | | Abundance of female *Ae. aegypti* mosquitoes | |
| AeAb_1_ | | Subsample of abundance of female *Ae. aegypti* mosquitoes taken from BGS traps | |
| Mo | | Month of collection | |
| Loc | | Location (study site) | |
| Nt | | Neighbourhood type (urban or peri-urban) | |
| Tr | | Trapping method (BG sentinel trap (BGS), Prokopack indoor (PPK-IN), and Prokopack outdoor (PPK-OUT)) | |
| Tmp | | Temperature as measured from INAMHI weather stations | |
| Tmp_1_ | | Temperature as measured from data loggers | |
| Ra_1_ | | Total rainfall from 14-8 days before sampling | |
| Ra_2_ | | Total rainfall from 21-15 days before sampling | |
| Ra_3_ | | Total rainfall from 22-28 days before sampling | |
| Ra_1,2,3_ | | Total rainfall from 8-28 days before sampling | |
| Hum | | Relative humidity as measured from data loggers | |
| Dt | | Date of sampling | |
| Nh | | Neighbourhood ID | |
| Hs | | House ID | |
| Col | | Collector ID (for Prokopack aspirations) or Trap ID (for BGS) | |

**RESULTS**

The two most abundant species were *Ae. aegypti* (24.73%) and *Culex quinquefasciatus* (68.15%) (Table B). Other mosquitoes collected included *Ae. angustivittatus* (0.2%)*, Anopheles pseudopunctipennis* (0.08%)*, Limatus durhami* (1.83%)*, Psorophora ferox* (0.75%)*, Aedes spp.* (0.68%)*, Anopheles spp.* (0.6%)*, Sabethes spp.* (0.08%)*,* and *Wyeomyia spp.* (0.25%) (Table B). A small proportion of mosquitoes (2.66%) could not be identified due to damage or loss of diagnostic features (Table B). Due to morphological similarities such as the golden-brownish colour of the scales, some of the *Ae. angustivittatus* may have been misidentified as *Cx. quinquefasciatus*.

Most *Ae. aegypti* individuals were collected by Prokopack indoor aspirations (49.79%) followed by BGS collections (36.6%) and Prokopack outdoor aspirations (13.59%) (Table B). Most *Cx. quinquefasciatus* were collected by Prokopack aspirations inside (49.72%) and in the outdoor area around houses (41.52%), followed by BGS (8.76%).

**Table B. Abundance of mosquitoes collected with BG-Sentinel (BGS) traps and Prokopack (PPK) aspirations in Portoviejo and Quinindé between November 2016 and April 2017**. Mosquitoes are broken down by sex (♂ = males, ♀= females), with females further split by blood feeding status. PPK aspirations were carried out inside houses and in the outdoor area for 10 minutes at each house area, while BGS collections were carried out outdoors for approximately 9 hours during the day.

|  | **Trapping methods** | | | | | | | | |  |  |  |  |
| --- | --- | --- | --- | --- | --- | --- | --- | --- | --- | --- | --- | --- | --- |
|  | **BGS traps** | | | **PPK aspirators** | | | | | | **Total counts** | | | |
|  |  |  |  | **Indoors** | | | **Outdoors** | | |  | | | |
| **Species** | ♀  fed | ♀  unfed | ♂ | ♀  fed | ♀  unfed | ♂ | ♀  fed | ♀  unfed | ♂ | Total  ♀  fed | Total  ♀ unfed | Total  ♂ | **Grand**  **Total** |
| *Aedes aegypti* | 68 | 197 | 96 | 242 | 82 | 167 | 31 | 33 | 70 | 341 | 312 | 333 | **986** |
| *Aedes angustivittatus* | 0 | 0 | 0 | 2 | 1 | 0 | 3 | 2 | 0 | 5 | 3 | 0 | **8** |
| *Aedes spp.* | 0 | 11 | 0 | 2 | 2 | 0 | 1 | 10 | 1 | 3 | 23 | 1 | **27** |
| *Anopheles pseudopunctipennis* | 0 | 0 | 0 | 2 | 1 | 0 | 0 | 0 | 0 | 2 | 1 | 0 | **3** |
| *Anopheles spp.* | 3 | 0 | 0 | 6 | 0 | 6 | 4 | 1 | 4 | 13 | 1 | 10 | **24** |
| *Culex quinquefasciatus* | 42 | 64 | 132 | 342 | 274 | 735 | 312 | 207 | 609 | 696 | 545 | 1476 | **2717** |
| *Limatus durhami* | 0 | 25 | 0 | 1 | 3 | 0 | 6 | 38 | 0 | 7 | 66 | 0 | **73** |
| *Psorophora ferox* | 2 | 20 | 0 | 1 | 2 | 0 | 0 | 4 | 1 | 3 | 26 | 1 | **30** |
| *Sabethes spp.* | 0 | 2 | 0 | 0 | 0 | 0 | 0 | 1 | 0 | 0 | 3 | 0 | **3** |
| *Wyeomyia spp.* | 0 | 5 | 0 | 0 | 2 | 0 | 0 | 3 | 0 | 0 | 10 | 0 | **10** |
| Unidentified | 10 | 9 | 28 | 0 | 13 | 12 | 19 | 0 | 15 | 29 | 22 | 55 | **106** |
|  |  |  |  |  |  |  |  | **Total** | | **1099** | **1012** | **1876** | **3987** |

**Table C. Abundance of mosquitoes collected with BG-Sentinel (BGS) traps and Prokopack (PPK) aspirations in Portoviejo and Quinindé between November 2016 and April 2017**. Mosquito species are broken down by period of collection and trapping method. PPK aspirations were carried out inside houses and in the outdoor area for 10 minutes at each house area, while BGS collections were carried out outdoors for approximately 9 hours during the day.

|  | **Collection Periods** | | | | | | | | | |  | | | **Total counts** | | | | |
| --- | --- | --- | --- | --- | --- | --- | --- | --- | --- | --- | --- | --- | --- | --- | --- | --- | --- | --- |
|  | 2016 | | | 2017 | | | | | | | | | |  |  |  |  |  |
|  | November | | | January | | | March | | | April | | | | Total per coll. period | | | | **Grand Total** |
| Species | BGS | PPK-IN | PPK-OUT | BGS | PPK-IN | PPK-OUT | BGS | PPK-IN | PPK-OUT | BGS | | PPK-IN | PPK-OUT | Nov./16 | Jan./17 | Mar./17 | Apr./17 |  |
| *Aedes aegypti* | 70 | 128 | 68 | 42 | 91 | 19 | 144 | 145 | 28 | 105 | | 127 | 19 | 266 | 152 | 317 | 251 | **986** |
| *Aedes angustivittatus* | 0 | 0 | 0 | 0 | 1 | 1 | 0 | 2 | 3 | 0 | | 0 | 1 | 0 | 2 | 5 | 1 | **8** |
| *Aedes* spp*.* | 0 | 0 | 0 | 0 | 0 | 0 | 5 | 4 | 2 | 6 | | 0 | 10 | 0 | 0 | 11 | 16 | **27** |
| *Anopheles pseudopunctipennis* | 0 | 3 | 0 | 0 | 0 | 0 | 0 | 0 | 0 | 0 | | 0 | 0 | 3 | 0 | 0 | 0 | **3** |
| *Anopheles* spp*.* | 0 | 6 | 0 | 0 | 1 | 1 | 0 | 1 | 0 | 3 | | 4 | 8 | 6 | 2 | 1 | 15 | **24** |
| *Culex quinquefasciatus* | 19 | 146 | 83 | 33 | 34 | 35 | 33 | 185 | 98 | 16 | | 162 | 301 | 248 | 102 | 316 | 479 | **1145** |
| *Culex* spp*.* | 6 | 212 | 92 | 13 | 253 | 145 | 31 | 132 | 91 | 87 | | 227 | 280 | 310 | 411 | 254 | 594 | **1569** |
| *Limatus durhami* | 0 | 0 | 0 | 1 | 0 | 3 | 4 | 1 | 10 | 2 | | 2 | 10 | 0 | 4 | 15 | 14 | **33** |
| *Limatus* spp*.* | 0 | 0 | 0 | 8 | 0 | 4 | 2 | 1 | 4 | 8 | | 0 | 13 | 0 | 12 | 7 | 21 | **40** |
| *Psorophora ferox* | 0 | 0 | 0 | 1 | 0 | 0 | 2 | 0 | 3 | 18 | | 3 | 2 | 0 | 1 | 5 | 23 | **29** |
| *Sabethes* spp*.* | 0 | 0 | 0 | 0 | 0 | 0 | 1 | 0 | 0 | 1 | | 0 | 1 | 0 | 0 | 1 | 2 | **3** |
| *Wyeomyia* spp*.* | 0 | 0 | 0 | 0 | 0 | 0 | 4 | 2 | 3 | 1 | | 0 | 0 | 0 | 0 | 9 | 1 | **10** |
| Unknown | 0 | 1 | 1 | 38 | 1 | 2 | 4 | 23 | 35 | 5 | | 0 | 0 | 2 | 41 | 62 | 5 | **110** |
|  |  |  |  |  |  |  |  |  |  |  | | **Total** |  | **835** | **727** | **1003** | **1422** | **3987** |

**Table D. Summary table of statistical significance of explanatory variables tested for association with *Ae. aegypti* female abundance.** Significance values for each of the explanatory variables from the fitted models. Values of chi-square (*X^2^*)*,* degrees of freedom (df), and p-values for each of the covariates tested are shown. Bold values with an asterisk (*****) indicate significant terms. Fixed effects with a double S symbol (§) indicate the interaction term. “NA” indicates “not applicable” values for which single term significance was not possible because of their involvement in significant interaction terms. The letter “w” means week.

| **Explanatory variables** | ***X^2^*** | **df** | ***p*-value** |
| --- | --- | --- | --- |
| **Month of collection** | 10.11 | 3 | **0.02*** |
| **Canton** | NA | NA | NA |
| **Neighbourhood type** | 8.60 | 1 | **<0.01*** |
| **Trap type** | NA | NA | NA |
| **Temperature** | 0.01 | 1 | 0.94 |
| **Rain 1w ago** | 0.92 | 1 | 0.34 |
| **Rain 2w ago** | 0.62 | 1 | 0.43 |
| **Rain 3w ago** | 5.07 | 1 | **0.02*** |
| **Canton: Neighbourhood type §** | 0.05 | 1 | 0.82 |
| **Canton: Trap Type §** | 19.83 | 2 | **<0.001*** |
| **Canton: Month §** | 3.77 | 3 | 0.29 |
| **Neighbourhood type: Trap type §** | 0.83 | 2 | 0.66 |
| **Temperature: Canton §** | 0.12 | 1 | 0.73 |

**Table E. Estimated mean abundance of *Ae. aegypti* females**. Mean values are given for each month of collection neighbourhood type, and canton and trap type combination, with the corresponding 95% CI of the lower and upper limits. Values for each of the three trapping methods, BG-Sentinel traps (BGS) and indoor Prokopack aspirations (PPK-IN) and outdoor (PPK-OUT) are given too.

| Covariates | Covariate levels | | Mean | 95% CI | |
| --- | --- | --- | --- | --- | --- |
|  |  |  |  | Lower lim. | Upper lim. |
| Month | November 2016 | | 0.45 | 0.19 | 1.07 |
|  | January 2017 | | 0.44 | 0.21 | 0.93 |
|  | March 2017 | | 1.37 | 0.81 | 2.31 |
|  | April 2017 | | 1.17 | 0.47 | 2.89 |
| Neighbourhood type | Urban | | 1.12 | 0.82 | 1.53 |
|  | Peri-urban | | 0.53 | 0.37 | 0.75 |
| Canton and Trap type | Portoviejo | BGS | 0.97 | 0.56 | 1.68 |
|  |  | PPK-IN | 1.87 | 1.15 | 3.05 |
|  |  | PPK-OUT | 0.32 | 0.17 | 0.62 |
|  | Quinindé | BGS | 1.30 | 0.75 | 2.27 |
|  |  | PPK-IN | 0.64 | 0.35 | 1.18 |
|  |  | PPK-OUT | 0.44 | 0.23 | 0.84 |

**Table F. Summary table of significance of variables tested for microclimatic association with *Ae. aegypti* female abundance.** Significance values for each of the explanatory variables from the fitted models. Values of chi-square (*X^2^*)*,* degrees of freedom (df), and p-values for each of the covariates tested are shown. Bold values with an asterisk (*****) indicate significant terms. Fixed effects with a double S symbol (§) indicate the interaction term. “NA” indicates “not applicable” values for which single term significance was not possible because of their involvement in significant interaction terms. The letter “w” means week.

| **Explanatory variables** | *X^2^* | df | *p*-value |
| --- | --- | --- | --- |
| **Month of collection** | 12.84 | 2 | **<0.01*** |
| **Canton** | 2.12 | 1 | 0.15 |
| **Neighbourhood type** | 2.62 | 1 | 0.11 |
| **Humidity** | 0.75 | 1 | 0.39 |
| **Temperature** | 2.10 | 1 | 0.15 |
| **Rain 1w ago** | 0.33 | 1 | 0.56 |
| **Rain 2w ago** | 0.10 | 1 | 0.75 |
| **Rain 3w ago** | 8.68 | 1 | **<0.01*** |
| **Canton: Neighbourhood type §** | 0.16 | 1 | 0.69 |
| **Canton: Temperature §** | 0.83 | 1 | 0.36 |
| **Canton: Month §** | 5.60 | 1 | 0.06 |
| **Canton: Humidity §** | 1.71 | 2 | 0.19 |
| **Temperature: Humidity §** | 2.34 | 1 | 0.13 |

**Table G. Summary table of statistical significance of explanatory variables tested for association with dengue incidence. Analysis based on a subset of incidence data corresponding to the timing of *Aedes* vector surveillance carried out in each canton between November 2016 and April 2017.** Values of chi-square (*X^2^*)*,* degrees of freedom (df), and *p*-values for each of the predictors tested are shown. Bold values with an asterisk (*) indicate significant terms. “NA” indicates “not applicable” values for which single term significance was not possible because of their involvement in significant interaction terms.

| **Lag periods** | **Explanatory variables** | ***X^2^*** | **df** | ***p*-value** |
| --- | --- | --- | --- | --- |
| 0 week lag | Canton | 51.85 | 1 | **< 0.001*** |
|  | BG-Sentinel trap | 6.10 | 1 | **< 0.05*** |
|  | Indoor Prokopack aspiration | 29.14 | 1 | **< 0.001*** |
|  | Outdoor Prokopack aspiration | 26.40 | 1 | **< 0.001*** |
| 1 week lag | Canton | 29.42 | 1 | **< 0.001*** |
|  | BG-Sentinel trap | 1.95 | 1 | 0.16 |
|  | Indoor Prokopack aspiration | 16.65 | 1 | **< 0.001*** |
|  | Outdoor Prokopack aspiration | 23.90 | 1 | **< 0.001*** |
| 2 week lag | Canton | 6.98 | 1 | **< 0.01*** |
|  | BG-Sentinel trap | 0.17 | 1 | 0.68 |
|  | Indoor Prokopack aspiration | 0.08 | 1 | 0.78 |
|  | Outdoor Prokopack aspiration | 0.17 | 1 | 0.68 |
